# Notoginsenoside R1 Facilitated Wound Healing in High-Fat Diet/Streptozotocin-Induced Diabetic Rats

**DOI:** 10.1155/2022/2476493

**Published:** 2022-01-13

**Authors:** Guangzhao Cao, Changpei Xiang, Rui Zhou, Yi Zhang, He Xu, Hongjun Yang, Jingjing Zhang

**Affiliations:** ^1^Institute of Chinese Materia Medica, China Academy of Chinese Medical Sciences, Beijing 100700, China; ^2^Experimental Research Center, China Academy of Chinese Medical Sciences, Beijing 100700, China

## Abstract

Diabetic ulcers bring about high morbidity and mortality in patients and cause a great economic burden to society as a whole. Since existing treatments cannot fulfil patient requirements, it is urgent to find effective therapies. In this study, the wound healing effect of topical notoginsenoside R1 (NR1) treatment on diabetic full-thickness wounds in type II diabetes mellitus (T2DM) was induced by the combination of a high-fat diet and streptozotocin (STZ) injection. NR1 significantly increased the wound closure rate, enhanced extracellular matrix (ECM) secretion, promoted collagen growth, increased platelet endothelial cell adhesion molecule-1 (CD31) expression, and decreased cleaved caspase-3 expression. RNA-Seq analysis identified ECM remodeling and inflammation as critical biological processes and Timp1 and Mmp3 as important targets in NR1-mediated wound healing. Further experiments showed that NR1-treated wounds demonstrated higher expression of tissue inhibitor of metalloproteinase 1 (TIMP1) and transforming growth factor-*β*1 (TGF*β*1) and lower expression of matrix metallopeptidase 9 (MMP9), matrix metallopeptidase 3 (MMP3), interleukin-1*β* (IL-1*β*), and interleukin-6 (IL-6) than diabetic wounds. These investigations promote the understanding of the mechanism of NR1-mediated diabetic wound healing and provide a promising therapeutic drug to enhance diabetic wound healing.

## 1. Introduction

Diabetic ulcers are serious complications that may lead to amputation in diabetic patients [1, 2]. Insufficient blood supply, impaired angiogenesis, and associated infection have been identified as the major reasons to ulcers in diabetic patients [3]. Studies have shown that nearly 25% of diabetic patients will progress to diabetic ulcers, which seriously threatens their health [4]. Wound healing in diabetes is associated with a series of pathological processes [5], such as oxidative stress [6], persistent inflammation [7], and apoptosis [8], and a decrease in extracellular matrix (ECM) secretion, resulting in nonunion of diabetic wounds [9]. Furthermore, macrophages and neutrophilic granulocytes aggregate slowly in the persistent inflammatory stage and correspond to increased inflammation in diabetic ulcer patients [10, 11], which is different from the inflammatory stage of normal wounds. At this time, the phagocytosis ability of leucocytes is impaired, and the existence time of inflammatory cytokines in wounds is prolonged, which inhibits the formation of granulation tissue and wound healing [12]. Meanwhile, abnormal cell apoptosis further prevents normal wound healing [8]. In addition, insufficient angiogenesis is a key factor in delayed diabetic wound healing [5, 13]. The ECM is expressed during wound healing and is essential for tissue repair, and its abnormality mainly affects the remodeling and proliferation stages of wound healing [9].

Current treatments include debridement [14], wound dressings [15], negative pressure wound therapy (NPWT) [16], and epidermal growth factor (EGF) [17]. However, there are some limitations to these treatments. For example, debridement often requires local infiltration with a significant risk of complications [18]. NPWT is a major clinical treatment, but the good results of NPWT can only be achieved when using a debridement to remove all devitalized, necrotic, and infected tissues [19]. The application of wound dressings depends on the type and severity of the wounds, as well as the condition of the patients [20]. Above all, these treatments are still unable to fulfill the patients' requirements and remain a huge challenge for diabetic wound healing. Therefore, it is important to find effective and convenient therapeutic strategies for diabetic wounds.

Recently, increasing evidence has demonstrated that traditional Chinese medicine (TCM) is effective and convenient for diabetic ulcer treatments. Our previous research indicated that topical treatment of the Huangbai line enhanced the key antioxidant regulator Nrf2 and its downstream antioxidant target genes, which increased wound closure and the synthesis of ECM and collagen while decreasing cell apoptosis and oxidative damage, accelerating wound healing in diabetes. As a major bioactive component from Coptis Salisb. (rhizome), berberine promoted wound healing in both type I diabetes and type II diabetes, which decreased oxidative damage, cell apoptosis, and ECM remodeling through thioredoxin reductase 1/c-Jun N-terminal kinase (TrxR1/JNK) signaling [21, 22]. Panax notoginseng and its main bioactive components exhibit pharmacological activity in various diseases, such as cancer, hypoglycemic effects, neuroprotection, and diabetes complications [23–25]. As one of the most important active components extracted from Panax notoginseng Notoginsenoside R1 (NR1) [26], it demonstrated an effective effect on various diabetic complications including diabetic nephropathy [27] and diabetic encephalopathy [28]. Relevant studies have reported that Panax Notoginseng Saponins (PNS) can alleviate skin ulcers in diabetic rats by reducing the expression of endothelin-1 (ET-1) and tumor necrosis factor-*α* (TNF-*α*) [29, 30]. However, the effect of NR1 on diabetic ulcers has not been reported, and the mechanism of NR-mediated wound healing remains unknown. In this study, the effect of topical NR1 treatment on type II diabetic wounds established by a high-fat diet and streptozotocin (STZ) injection combined with full-thickness wounds was systematically investigated by using RNA Sequencing (RNA-Seq) technology. Topical treatment of NR1 provides a convenient way to accelerate wound healing and allow NR1 to concentrate to the local wound site and avoid vascular dysfunction-induced absorption problems, offering a new treatment for diabetic ulcers.

## 2. Materials and Methods

### 2.1. Animals and Model Establishment

Sprague–Dawley rats that were adult male rats with weights between 160 and 180 g were supplied by the Beijing Huafukang Biotechnology Co., Ltd. (license number: SCXK (BJ) 2014-0004). All procedures in the experiment were conducted following the animal care and rules of the Institute of Chinese Materia Medica, China Academy of Chinese Medical Sciences. These rats were housed in an animal room with a 12-hour light/dark cycle environment and access to food and water continued to be freely available for 3 days. After adaptive feeding for 3 days, rats had free access to water and a high-fat diet for 4 weeks. The composition of high-fat diet is shown in Table 1. Streptozotocin (STZ, 35 mg/kg) was injected intraperitoneally into rats to induce diabetes and fasting for 12 h before the establishment of the diabetic model. For STZ-induced diabetes, the fasting blood glucose levels of rats randomly higher than 16.7 mmol/L but lower than 33.3 mmol/L were supposed to be diabetes. The diabetic rats were anesthetized with sodium pentobarbital, and two rubber rings (diameter, 2 cm) were sutured onto the skin of the diabetic rats. Then, two wounds that were 2 cm in diameter were symmetrically created on the rats' dorsum, with the left side rubbed by physiological saline and the right side rubbed by drugs [31]. Diabetic rats were divided into three groups according to a random number, and the rats received different treatments: physiological saline (hfdSTZ), NR1 (hfdSTZ+NR1, 0.038 mg/cm^2^), and rhEGF (hfdSTZ+rhEGF, 40 IU/cm^2^). All rats received topical therapy once per day for 15 consecutive days. After 14 days of treatment, fasting blood glucose levels were measured again. It is also noteworthy that in our experiment, the rats with fasting blood glucose levels between 16.7 mmol/L and 33.3 mmol/L were included. Representative photos of wound areas were taken at 0, 6, 9, 12, and 15 days during the treatment, and the wound closure was measured using ImageJ software, after which the wound closure rate was calculated.

### 2.2. Two-Photon Microscope- (SHG-) Based Collagen Growth Observation

First, collagen growth at the edge of the wounds was investigated in the rats by a two-photon microscope (SHG) after anesthetizing them with sodium pentobarbital.

### 2.3. Hematoxylin and Eosin (HE) Staining and Masson's Trichrome Staining

The skin tissues were placed into a new 4% paraformaldehyde solution at room temperature for 3 days. Then, the paraffin-embedded tissues were cut into sections with a thickness of 6 *μ*m for HE and Masson's trichrome staining. The stained slide was observed using a panoramic Digital Slide Scanner.

### 2.4. RNA-Seq Analysis

RNA extraction from the skin tissue was conducted with TRIzol reagent, and its integrality was measured. After quantification, RNA was then used to construct cDNA libraries. The sequencing process was completed according to previous protocols on an Illumina HiSeq 4000 platform, which was finished by Novogene Bioinformatics Technology Co., Ltd. (Beijing, China). Raw data for NR1-mediated wounds have been uploaded to the https://www.ncbi.nlm.nih.gov/sra/PRJNA757790. Differentially expressed genes (DEs) were also defined by using log2-fold change and *p* value analysis through the EBSeq algorithm, followed by a volcano plot to show the overall distribution. Then, genes with different expression patterns were grouped by hierarchical clustering. GO and KEGG enrichment analyses of DEs were performed using the DAVID, and the items were considered significant if the *p* value was less than 0.05. The biological processes and pathways associated with diabetic ulcers are shown in the bubble chart. Finally, a network of DEs and the enriched GO terms was then constructed via STRING and Cytoscape software.

### 2.5. Enzyme-Linked Immunosorbent Assay (ELISA)

Skin tissue homogenate was prepared for the following measurements. Levels of interleukin-1*β* (IL-1*β*), matrix metallopeptidase 9 (MMP9), interleukin-6 (IL-6), transforming growth factor-*β*1 (TGF-*β*1), matrix metallopeptidase 3 (MMP3), and tissue inhibitor of metalloproteinase 1 (TIMP1) were measured by ELISA. All steps were performed according to the manufacturer's instructions. Briefly, the sample was added to the ELISA plate coated with the monoclonal antibody and incubated for 1 h. Then, the biotinylated-labeled secondary antibody was added to the plate for 1 h. Next, the TMB substrate solution was added to the plate for 20 min before stopping the reaction with the stop solution. Then, the absorbance value was measured with a microplate reader (Molecular Devices, USA) at 450 nm.

### 2.6. Immunohistochemistry

Briefly, the skin tissues were rehydrated with H_2_O_2_ after dewaxing. Sections were loaded with the primary antibodies overnight. Secondary antibody incubation was followed by incubation for 10 min with streptavidin peroxidase. DAB (1x) was applied as the chromogen to incubate with the sections for 10 min at room temperature and then dyed by hematoxylin counterstain. Photos were captured under a light microscope and counted using ImageJ software. The antibodies used were as follows: TGF-*β*1 (sc-130348, Santa), MMP9 (ab76003, Abcam), platelet endothelial cell adhesion molecule-1 (CD31) (A0378, Abclonal), and cleaved caspase-3 (9664S, CST 9664S).

### 2.7. Statistical Analysis

Differences in data obtained in our manuscript were analyzed by one-way ANOVA, and all values are expressed as the mean ± S.D. *p* < 0.05 means that the difference between tested groups is statistically significant.

## 3. Results

### 3.1. Topical NR1 Treatment Enhanced the Healing of Diabetic Wounds

**Figure 1:**
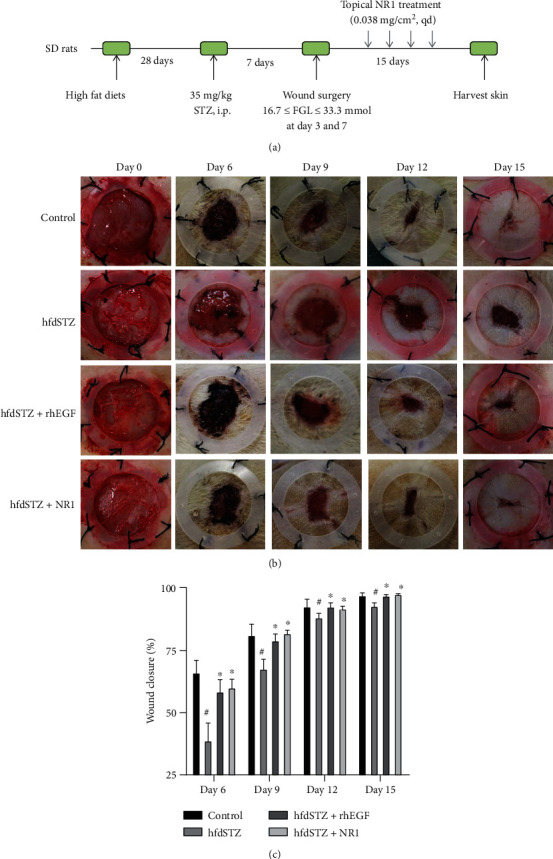
Topical NR1 treatment significantly promoted the wound closure rate in diabetic rats. (a) Schematic illustration showing the establishment of the diabetic ulcer model and treatment with NR1 for 15 days. (b) Representative images of diabetic wounds at Days 6, 9, 12, and 15. (c) The wound closure rate was quantitated by ImageJ software (*n* = 7-10). Data are presented as the mean ± SD; ^#^*p* < 0.05 vs. control; ^∗^*p* < 0.05 vs. hfdSTZ.

To investigate the effect of NR1 on diabetic wounds, the wound closure rate was evaluated. The hdfSTZ group showed a lower wound closure rate than the control group (Figures 1(b) and 1(c)). NR1 significantly increased the wound closure rate at Days 6, 9, 12, and 15 compared to the hfdSTZ group, which was equivalent to that in the hfdSTZ+rhEGF group. Thus, topical NR1 and rhEGF treatment enhanced wound healing in T2DM.

### 3.2. NR1 Decreased Cleaved Caspase-3 and Increased ECM Secretion and CD31 Expression in Diabetic Wounds

**Figure 2:**
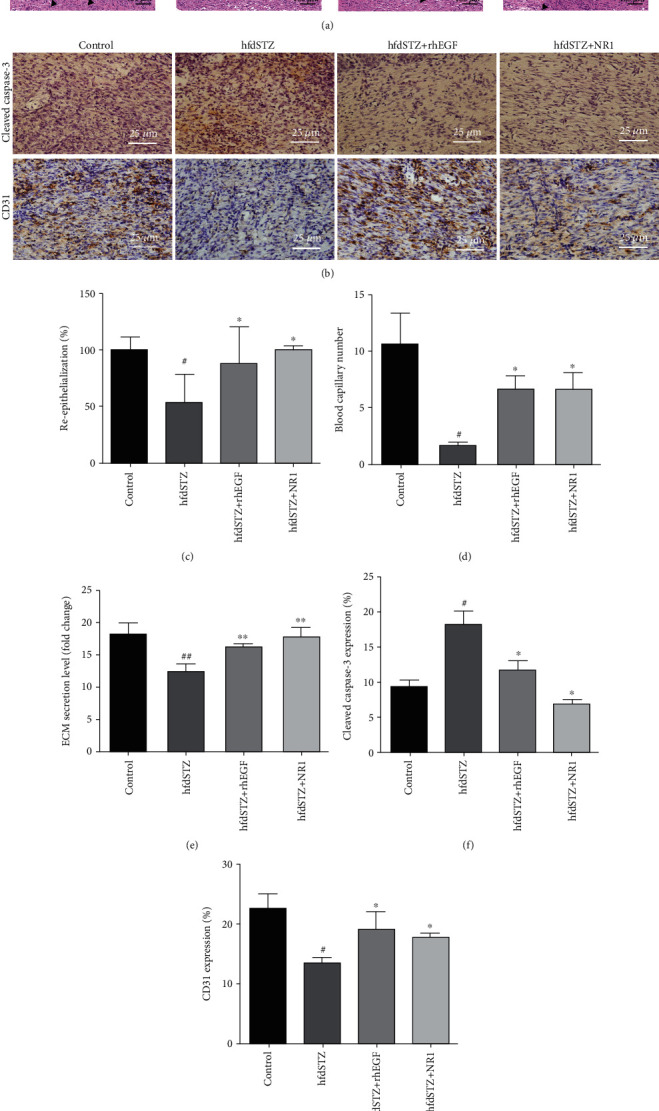
Effect of NR1 treatment on ECM secretion and the expression of cleaved caspase-3 and CD31 in diabetic wounds. (a) HE staining. (b) Immunohistochemical staining for cleaved caspase-3 (*n* = 4). Scale bar: 25 *μ*m; immunohistochemical staining for CD31 (*n* = 3-4). Scale bar: 25 *μ*m. (c) Quantified re-epithelialization (*n* = 3). (d) Quantified blood capillary (*n* = 3). (e) Quantified ECM secretion level (*n* = 3). (f) Quantified immunohistochemical staining of cleaved caspase-3 (*n* = 4). (g) Quantified immunohistochemical staining of CD31 (*n* = 3-4). Data are presented as the mean ± SD. Significance: ^#^*p* < 0.05 vs. control and ^∗^*p* < 0.05 vs. hfdSTZ.

To investigate the effect of NR1 on ECM secretion, apoptosis and angiogenesis in diabetic ulcer rats, HE staining, cleaved caspase-3, and CD31 expression were evaluated. The results revealed that the epithelial tissue (black arrow in Figures 2(a) and 2(c)) in the diabetic wounds was significantly increased after NR1 or rhEGF treatment, whereas there was no significant repair in the hfdSTZ group (yellow arrow in Figure 2(a)). This further proved that re-epithelialization was accelerated in the hfdSTZ+NR1 group and hfdSTZ+rhEGF group. In addition, there was more blood capillary regeneration (black triangle in Figures 2(a) and 2(d)) in the hfdSTZ+NR1 group and hfdSTZ+rhEGF group than in the hfdSTZ group. Furthermore, there was lower secretion of ECM in the hfdSTZ group than in the control group at Day 15, but the secretion of ECM was significantly increased after treatment with NR1 and rhEGF (Figures 2(a) and 2(e)). In addition, as shown in Figures 2(f) and 2(g), an obvious increase in cleaved caspase-3 and a decrease in CD31 were observed in the hfdSTZ group compared to the control group, whereas NR1- and rhEGF-treated wounds displayed lower cleaved caspase-3 expression and higher CD31 expression. The immunochemical staining results identified that cleaved caspase-3 expression in the hfdSTZ+NR1 group and hfdSTZ+rhEGF group was lower than that in the hfdSTZ group, and CD31 staining was higher than that in the hfdSTZ group (Figure 2(b)). Thus, NR1 and rhEGF reduced apoptosis and promoted angiogenesis and ECM secretion in diabetic wounds.

### 3.3. RNA Sequencing to Identify the Possible Mechanism of NR1 in Diabetic Wounds

**Figure 3:**
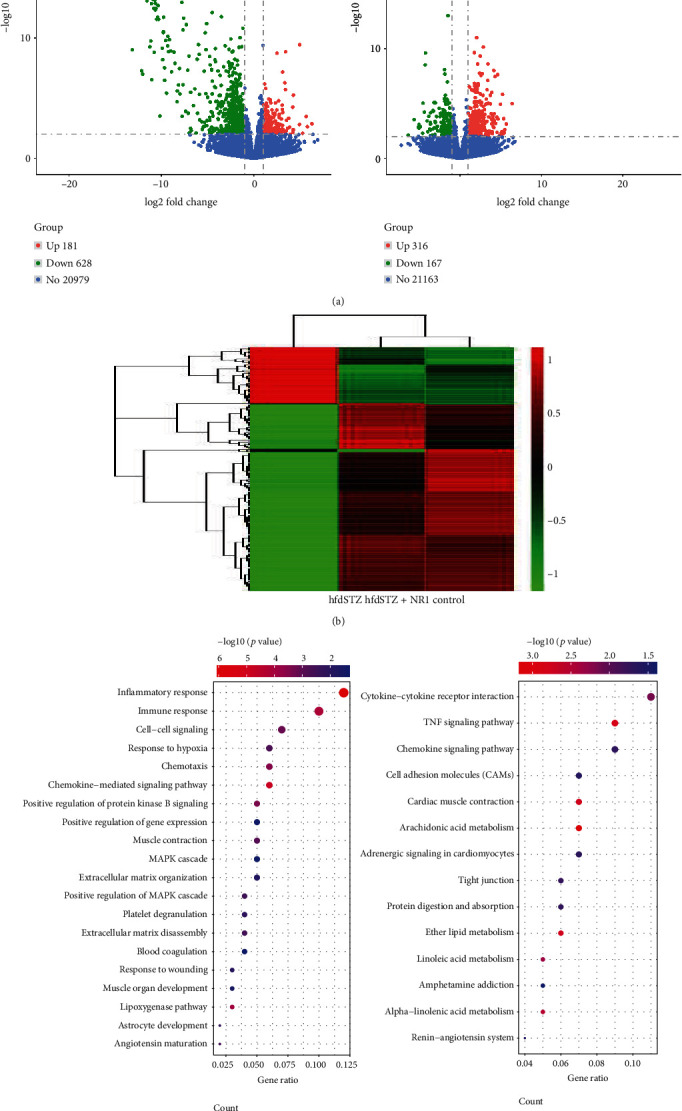
The global gene expression pattern of skin wounds. (a) Volcano map of the DEGs with upregulated genes (red) and downregulated genes (green); blue represents nondifferentially expressed genes. (b) The heatmap. (c) GO and KEGG pathway analysis of these DEGs; the horizontal axis represents the ratio of the number of differential genes and the total differential genes enriched in GO enrichment or KEGG pathway; the vertical axis represents GO or KEGG terms.

Compared to the control group, 809 DEGs were observed in the hfdSTZ group, with 181 upregulated genes and 628 downregulated genes. In contrast to the hfdSTZ group, 316 DEGs were significantly upregulated, and 167 genes were significantly downregulated in the hfdSTZ+NR1 group (Figure 3(a)). In addition, heatmap results indicated that the gene expression pattern was different between the hfdSTZ group and the NR1 group. The gene expression patterns of the hfdSTZ+NR1 group and the control group were similar (Figure 3(b)). To further understand the underlying biological processes and pathways, GO and KEGG enrichment analysis of DEGs between the hfdSTZ+NR1 and hfdSTZ groups was carried out (Figure 3(c)). Furthermore, these DEGs after NR1 treatment were enriched in biological processes and pathways such as the inflammatory response, response to hypoxia, chemotaxis, chemokine-mediated signaling pathways, extracellular matrix organization, TNF signaling pathway, tight junctions, and cell adhesion molecules.

### 3.4. Identification of Critical Biological Processes and Targets in NR1-Mediated Diabetic Wound Repair

**Figure 4:**
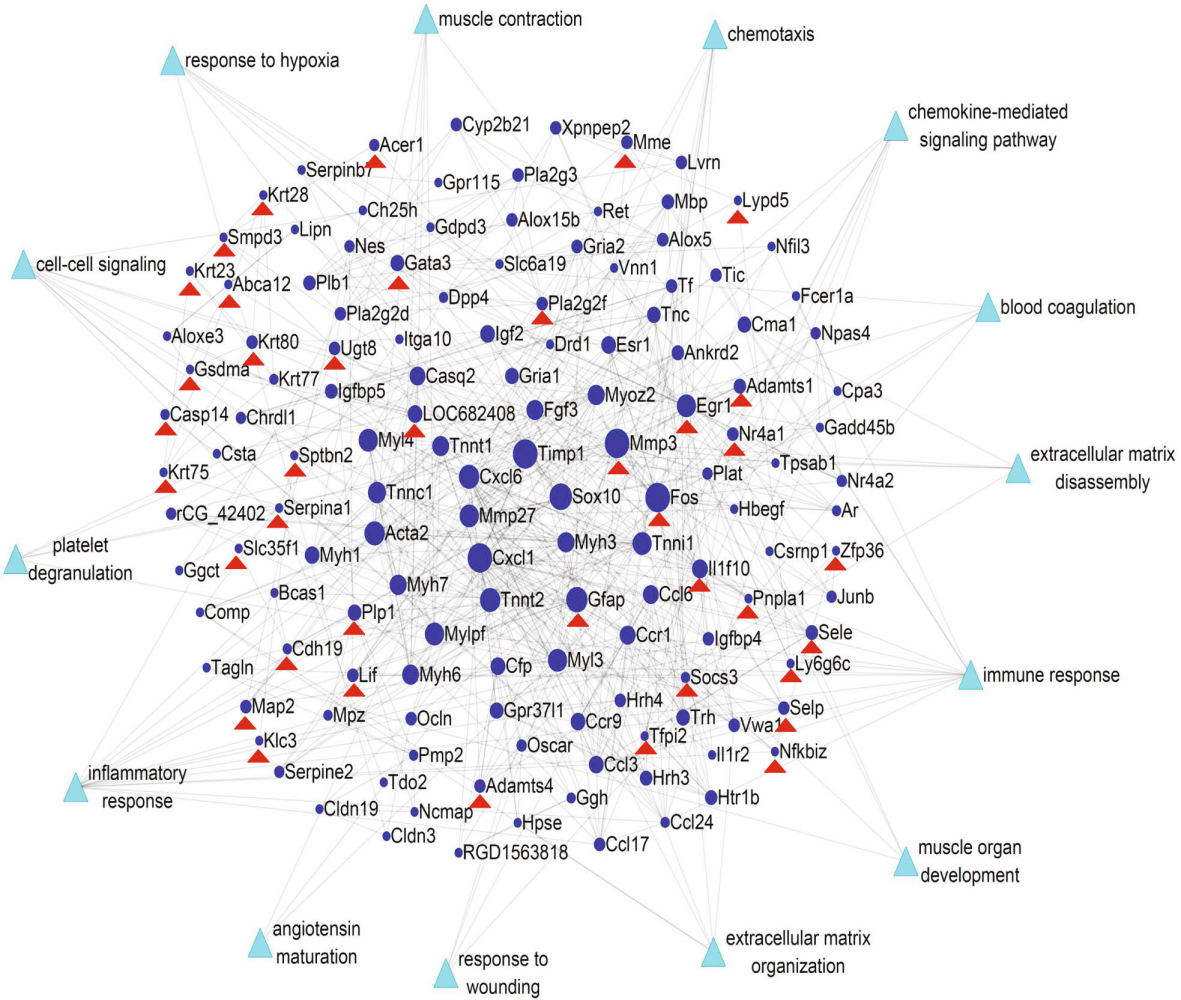
The network based on DEGs between the hfdSTZ group and hfdSTZ+NR1 group and biological processes associated with diabetic wound repair; red triangles represent DEGs in the hfdSTZ+NR1 group and hfdSTZ group; light blue triangles represent the biological processes associated with wound repair in diabetes.

A network of DEs and biological processes regulated by NR1, which is associated with diabetic wound repair, was constructed. As shown in Figure 4, we identified ECM-related processes and inflammation as critical processes, since the DESs were enriched in GO terms such as extracellular matrix disassembly, extracellular matrix organization, immune response, inflammatory response, chemotaxis, and chemokine-mediated signaling pathway. By analyzing the degree of the targets in this network, targets, such as Cxcl1, Fos, Timp1, and Mmp3, with the highest degree were identified as vital targets in NR1-mediated wound healing.

### 3.5. Verification of the Critical Biological Processes and Targets in NR1-Mediated Diabetic Wound Healing

**Figure 5:**
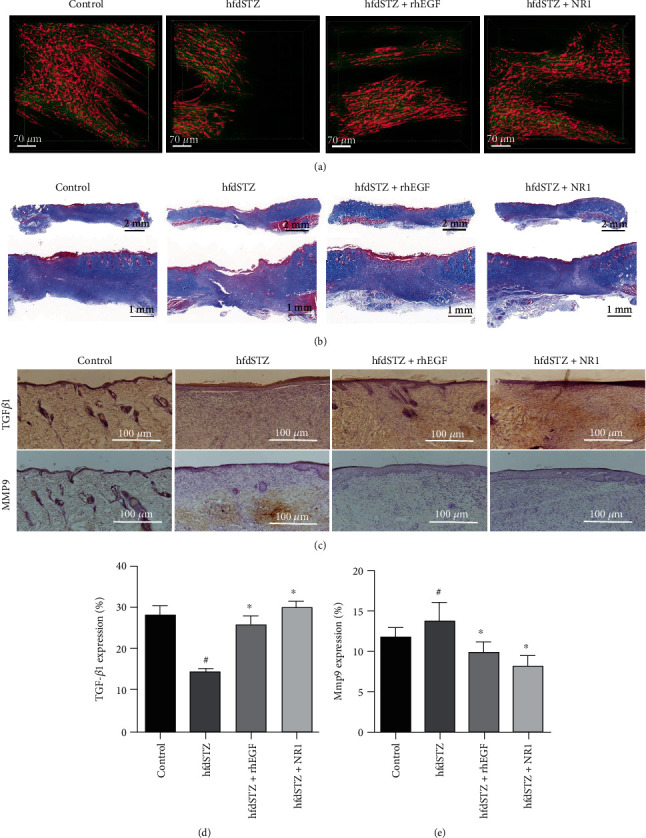
Topical NR1 treatment promoted collagen expression and maintained the generation and remodeling of ECM in diabetic rats. (a) Pink represents new collagen growth at the edge of diabetic wounds; green represents the background of Day 15. Scale bar: 70 *μ*m. (b) Masson staining. (c) Immunohistochemical staining for TGF-*β*1 and MMP9 (*n* = 4). Scale bar: 100 *μ*m; (d) Quantified immunohistochemical staining of TGF-*β*1 (*n* = 4). (e) Quantified immunohistochemical staining of MMP9 (*n* = 3) (*n* = 4). Data are presented as the mean ± SD. Significance: ^#^*p* < 0.05 vs. control and ^∗^*p* < 0.05 vs. hfdSTZ.

**Figure 6:**
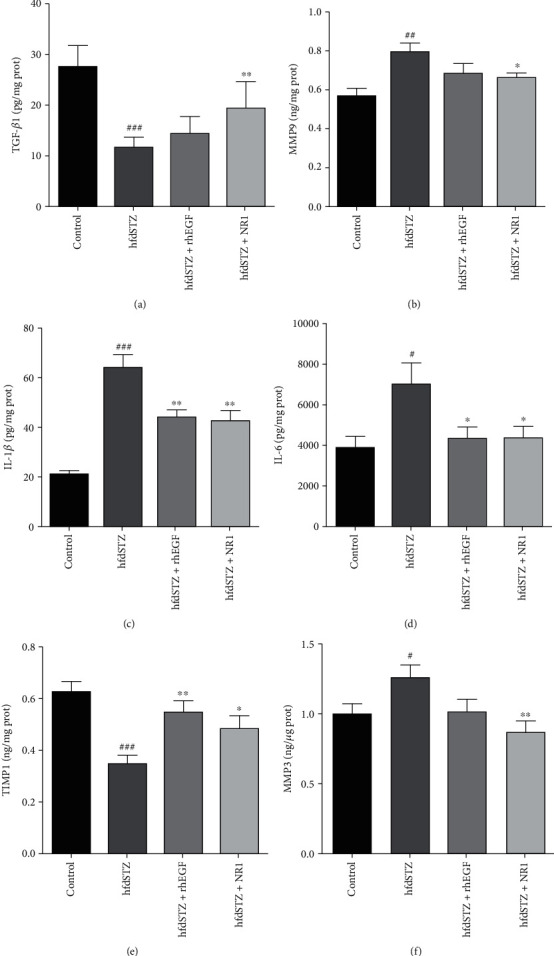
Effect of NR1 treatment on TGF-*β*1, MMP9, IL-1*β*, IL-6, TIMP1, and MMP3 in diabetic wounds. (a) ELISA results of TGF-*β*1 (*n* = 8). (b) ELISA results of MMP9 (*n* = 8). (c) ELISA results of IL-1*β* (*n* = 8). (d) ELISA results of IL-6 (*n* = 5-6). (e) ELISA results of TIMP1 (*n* = 8). (f) ELISA results of MMP3 (*n* = 6-8). Data are presented as the mean ± SD. ^#^*p* < 0.05, ^##^*p* < 0.01, and ^###^*p* < 0.001 vs. control and ^∗^*p* < 0.05 and ^∗∗^*p* < 0.01 vs. hfdSTZ.

To verify the critical biological processes, ECM synthesis and remodeling were evaluated by determining collagen growth and the expression of MMP9 and TGF-*β*1. Collagen synthesis was visualized using a two-photon microscope (SHG) and Masson's staining. The SHG results demonstrated that the collagen expression at the front edge of wounds was lower at Day 15 in hfdSTZ than in the control, whereas this was increased after topical treatment with NR1 and rhEGF (Figure 5(a)). Consistent with this result, Masson's trichrome staining showed that NR1 and rhEGF treatment increased the low collagen expression in diabetic wounds (Figure 5(b)). The immunochemical staining and ELISA results showed that NR1 increased TGF-*β*1 expression while decreasing MMP9 expression in diabetic wounds (Figures 5(c)–5(e), 6(a), and 6(b)). Taken together, NR1 promoted collagen expression and maintained ECM synthesis and remodeling in diabetic ulcer rats.

Since the DEGs after NR1 treatment were enriched in biological processes, such as inflammation, IL-1*β* and IL-6 were measured. The ELISA results demonstrated that NR1-treated wounds had lower expression of IL-1*β* and IL-6 than hfdSTZ-treated wounds (Figures 6(c) and 6(d)). The identified critical targets, such as tissue inhibitor of metalloproteinase 1 (TIMP1) and MMP3, were also confirmed by ELISA. As shown in Figures 6(e) and 6(f), the high level of MMP3 in the hfdSTZ group was significantly reduced by NR1 and rhEGF treatment. In addition, a low level of TIMP1 in diabetic wounds was increased by NR1 treatment compared with that in the hfdSTZ group. Thus, NR1 reduced inflammation and ECM degradation while enhancing collagen secretion, and TIMP1 and MMP3 were potential critical targets.

## 4. Discussion

In this study, a skin ulcer model was established by full-thickness wounds in type 2 diabetic rats to evaluate the effect of NR1 on diabetic wound healing. Furthermore, the possible mechanism of NR1 against diabetic ulcers was elucidated by using RNA-Seq technology. NR1 obviously enhanced the wound healing rate, promoted collagen expression, and increased CD31 expression while decreasing cleaved caspase-3 expression. By applying RNA-Seq technology, we identified extracellular matrix related processes and inflammation as critical biological processes and Timp1 and Mmp3 as important targets in NR1-mediated wound healing. Our further experiment confirmed the inhibitory effect of NR1 on ECM remodeling and inflammation, which was evidenced by low expression of MMP9, MMP3, IL-1*β*, and IL-6 and higher expression of TGF-*β*1 and TIMP1.

Numerous studies have indicated that wound healing in diabetes is due to apoptosis [32] and insufficient angiogenesis [5, 13]. In our study, we showed that NR1 decreased cleaved caspase-3 and increased CD31 expression in diabetic wounds, which indicated that topical NR1 treatment attenuated apoptosis, increased angiogenesis, and consequently enhanced wound healing in diabetic rats. Angiogenesis is a key factor in facilitating wound healing [33, 34]. Abnormal angiogenesis can result in impaired wound healing, and the mechanism may be related to the change in macrophage phenotype and failure to promote tissue repair [13]. CD31 was previously widely applied as an angiogenesis marker [35, 36]. Furthermore, abnormal apoptosis is associated with impaired wound healing, resulting in slow wound healing [8]. During the process of wound healing, apoptosis usually helps to remove inflammatory cells and is closely associated with the development of granulation tissue to scar tissue [37]. However, the accumulation of advanced glycation end products and the increase in ROS production caused mitochondrial damage, decreased expression of B-cell lymphoma-2 (Bcl-2), and increased expression of caspase in diabetic wounds. Therefore, the proliferation of fibroblasts is inhibited, and wound healing is delayed [8]. Cleaved caspase-3 acts as a major executive caspase in apoptosis, and a relevant study found that cleaved caspase-3 activity is elevated in diabetic ulcer rats [22, 38–40]. These results reflected our results that diabetic ulcers were associated with the biological processes of apoptosis and angiogenesis.

Importantly, inflammation is relevant for wound repair and leads to decreased vascularization and tissue necrosis, which impair wound healing [41, 42]. IL-1*β*, a proinflammatory cytokine, is considered an indicator of inflammation and plays an important role in the impairment of chronic and nonhealing diabetic wounds [43–45]. A previous study showed that the wound healing rate can be accelerated through the marked downregulation of IL-1*β* in diabetic foot ulcer rats [46]. IL-6 is another inflammatory cytokine that is thought to be related to wound healing and a relevant study showed that the production of IL-6 in dermal tissue delayed the dermal wound healing response [47]. Furthermore, long-term diabetic ulcers are usually accompanied by less ECM regeneration and excessive degradation, which is characterized by low TGF-*β*1 and TIMP1 expression and high MMP3 and MMP9 levels. In our study, we showed that topical NR1 treatment increased TGF-*β*1 and TIMP1; decreased IL-1*β*, IL-6, and MMP3, and MMP9; and contributed to enhanced ECM secretion and wound healing. It is difficult for diabetic wounds to heal spontaneously because of abnormal remodeling and impairment of ECM synthesis [48]. TGF-*β*1 is a key regulator of the ECM in collagen synthesis [49, 50]. In addition, the degradation of the ECM is regulated by tissue inhibitors of TIMPs and MMPs, both to maintain homeostasis of the ECM, and diabetic ulcer patients often demonstrate overproduction of MMPs with low TIMPs [51–53]. TIMP1 is a glycoprotein belonging to the family of TIMPs [54]; it inhibits most MMPs and may help perform its normal function [55, 56]. Furthermore, TIMP1 also promotes cell proliferation and inhibits apoptosis [57]. A previous study found that increased MMP levels are associated with delayed wound healing [58]. MMP3 is a master controller of the matrix metalloproteinase family [59] and is able to activate a variety of matrix metalloproteinase precursors and participate in the degradation of the ECM and remodeling phases [60]. MMP9, a subtype of matrix metalloproteinase, is involved in the breakdown of extracellular matrix in normal physiological processes, such as tissue remodeling and metastasis [61]. These results demonstrated that the process of inflammation and the targets of TGF-*β*1, TIMP1, MMP3, and MMP9 play a key role in the progression of NR1 treatment in diabetic ulcers.

## 5. Conclusions

In summary, NR1 has therapeutic efficacy against diabetic ulcers by reducing inflammation and apoptosis, increasing angiogenesis and maintaining ECM remodeling. Importantly, our study provides clear evidence for the successful use of topical NR1 treatment for type II diabetic ulcers, and its molecular mechanism was explored. NR1 may have potential as a new, practical strategy for diabetic ulcer treatment.

## Figures and Tables

**Table 1:** Composition of the experimental high-fat diet.

Components	Mass ratio (g)
Protein (casein, L-cysteine)	230
Carbohydrate (dextrin, source)	330
Fat (soybean oil, lard)	367
Mineral and vitamin mixture	73
Antioxidant (TBHQ)	0.07
Energy (kcal/g)	5.5
% kcal from protein	15%
Carbohydrate	25%
Fat	60%
Total	100%

## Data Availability

All data in this study can be obtained from the appropriate authors.

## Abbreviations

NR1: Notoginsenoside R1
T2DM: Type II diabetes mellitus
STZ: Streptozotocin
ECM: Extracellular matrix
CD31: Platelet endothelial cell adhesion molecule-1
TIMP1: Tissue inhibitor of metalloproteinase 1
TGF-*β*1: Transforming growth factor-*β*1
MMP9: Matrix metallopeptidase 9
MMP3: Matrix metallopeptidase 9
IL-1*β*: Interleukin-1*β*
IL-6: Interleukin-6
NPWT: Negative pressure wound therapy
EGF: Epidermal growth factor
TCM: Traditional Chinese medicine
TrxR1: Thioredoxin reductase 1
JNK: c-Jun N-terminal kinase
PNS: Panax Notoginseng Saponins
ET-1: Endothelin-1
TNF-*α*: Tumor necrosis factor-*α*
RNA-Seq: RNA Sequencing
HE: Hematoxylin and eosin
DEs: Differentially expressed genes
ELISA: Enzyme-linked immunosorbent assay.

## Supplementary Materials

Supplementary 1 Tab. S1: composition of the experimental high-fat diet.

Supplementary 2 Tab. S2: gene expression of diabetic wounds treated with NR1 by using RNA-Seq analysis.
